# Comprehensive toolkit integrating lifestyle and clinical questionnaires with gut microbiota profiling via rectal swabs: application in intensive care cirrhotic patients

**DOI:** 10.1099/jmm.0.001964

**Published:** 2025-03-06

**Authors:** Julie Marin, Mohamed Ghalayini, Younes Kaoudji, Samira Dziri, Cecile Zylberfajn, Lorraine Blaise, Astrid Hoogvorst, Stephane Charpentier, Virginie Chaillou, Sylvie Beauchamp, Séverine Donneger, Nathalie Barget, Mathilde Touvier, Pierre Nahon, Roland Amathieu, Mathilde Lescat

**Affiliations:** 1 Université Sorbonne Paris Nord and Université Paris Cité, Inserm, IAME, F-93000 Bobigny, France; 2 Université Sorbonne Paris Nord and Centre hospitalier de Gonesse, Gonesse, France; 3 Service Microbiologie, AP-HP, Hôpitaux Universitaires de Paris Seine Saint Denis France, Paris, France; 4 Service de Réanimation, AP-HP, Hôpitaux Universitaires de Paris Seine Saint Denis, Paris, France; 5 Université Sorbonne Paris Nord and Service d’Hépatologie, AP-HP, Hôpitaux Universitaires de Paris Seine Saint Denis, Paris, France; 6 Centre de Ressources biologiques, AP-HP, Hôpitaux Universitaires de Paris Seine Saint Denis, Paris, France; 7 Université Sorbonne Paris Nord and Université Paris Cité, Inserm, EREN, F-93000 Bobigny, France; 8 Université Paris Cité, Inserm, Institut Cochin, F-75014 Paris, France; 9 Bacteriology Unit, Microbiology and Infectious Diseases Department, Institut de Recherche Biomédicale des Armées, 91220 Brétigny-sur-Orge, France; 10 CNR-LE Charbon, Institut de Recherche Biomédicale des Armées, 91220 Brétigny-sur-Orge, France and Aix Marseille Université, INSERM, SSA, MCT, Marseille, France

**Keywords:** clinical research, cirrhotic patients, gravity, gut microbiota, prediction of infections

## Abstract

**Introduction.** The study of gut microbiota is now an essential dimension in many clinical studies. For instance, microbiota diversity investigation can help us to better manage cirrhotic patients by the identification of markers of severity and the identification of possible sources of pathogens.

**Hypothesis/Gap Statement.** Conducting clinical research on gut microbiota for fragile patients in intensive care units, such as cirrhotic patients, poses significant challenges.

**Aim.** In this study, we developed a comprehensive toolkit for investigating gut microbiota in fragile patients using rectal swabbing combined with straightforward lifestyle and clinical questionnaires.

**Methodology*****.*** We applied this prospective approach to 49 well-phenotyped cirrhotic patients as a function of their compensation status (compensated patients with outpatients’ recruitment vs decompensated patients in intensive care units).

**Results*****.*** Our results, consistent with the literature, showed that liver function impairment is associated with lower microbiota diversity. Additionally, we monitored aerobic microbiota in decompensated cirrhotic patients, observing the invasion of extended spectrum beta-lactamase (ESBL)-producing *Escherichia coli* in the gut’s aerobic microbiota prior to severe infection caused by these pathogens.

**Conclusion.** We propose this pragmatic methodology for larger cohort studies, aiming to enhance the monitoring of immunocompromised patients by using microbiota analysis as a predictive tool for the severity of associated pathologies and the identification of agents responsible for severe infections.

## Code availability

The code (R language) used in this study to perform statistical analyses and figures has been deposited on GitHub https://github.com/j-marin/CIRCOM.git.

## Introduction

Cirrhosis is a chronic liver disease characterized by the accumulation of scar tissue that impairs the normal functioning of the liver. Cirrhosis, which has different aetiologies (viral, alcohol and obesity), is a major public health problem in France. The main causes of mortality are due to infectious complications, liver failure and hepatocellular carcinoma (joint complications or not) [1,2]. The severity of cirrhosis appeared closely linked to gut dysbiosis in microbiota, suggesting a role in disease progression [3]. The human microbiota, a diverse community of micro-organisms residing in our bodies, is a key area in health research. A multitude of studies have endeavoured to uncover the forces that shape the human microbiota, with genetics – spanning sex differences – emerging as a key player [4,5]. Mode of birth, age, diet and environmental exposures, such as medications (especially antibiotics), also hold significant sway [6,9]. Moreover, behaviours like physical activity contribute to the dynamic landscape of the microbiome [10]. Dysbiosis, characterized by imbalances in microbial composition, has also been linked to various diseases, including obesity and inflammatory bowel diseases in addition to cirrhosis [11,14]. Quantitative assessment of aerobic bacteria is also crucial for understanding severe infections. Indeed, imbalances in the gut microbiota are linked to infections, such as urinary tract infections caused by *Escherichia coli*, which correlate with elevated *Escherichia coli* quantities in the digestive tract pre-infection [15]. Moreover, multidrug-resistant organisms (MDROs) present a significant threat, requiring innovative approaches. Quantitative analysis could provide insights into MDRO dynamics within the microbiota, helping to understand infection mechanisms, to predict MDRO-associated infections and to monitor resistance patterns over time [16]. Understanding the interactions between the microbiota and pathogenic agents is of primary importance in the prevention and treatment of multiple diseases, paving the way for novel therapeutic strategies targeting microbiota modulation.

Considering these complementary aspects, cirrhosis emerges as an ideal model for investigating infections and MDRO in the context of dysbiosis. Indeed, dysbiosis is linked to the pathogenesis of cirrhosis, and bacterial infections play a pivotal role in cirrhosis decompensation [17]. Furthermore, among the primary severe bacterial infections, cirrhosis stands out as a key risk factor exacerbating the severity of *Escherichia coli* bacteraemia [18]. These findings have implications for cirrhosis management, aligning with prior research showing the effectiveness of interventions like prebiotics, probiotics, faecal transplantations and even antibiotics to manage dysbiosis or *Enterobacteriaceae* burden in improving liver function and reducing inflammation [3,19].

The analysis by sequencing of 16S rRNA gene or metagenomics of the microbiota of cirrhotic patients showed significant differences compared to healthy people, including decreased diversity (specially *Bacteroidetes*, *Ruminococcus*, *Roseburia*, *Veillonellaceae* and *Lachnospiraceae*), alongside with an increase in potentially harmful species belonging to the phyla *Fusobacteriota* and *Proteobacteria* (specially *Enterobacteriaceae*) and to the families *Enterococcaceae* and *Streptococcaceae* [18]. However, investigating the gut microbiota in cirrhotic patients poses challenges due to difficulties in obtaining high-quality samples using conventional total faecal sampling methods, particularly in severely ill patients requiring urgent antibiotic therapy. We previously published a study that defines an optimal protocol to study the microbiota diversity (total and aerobic). Here, we developed a pragmatic approach to study gut microbiota using 16S rRNA gene quantification and aerobic culturomics, applicable in various clinical settings such as intensive care or emergency units, where whole stool sampling is often impractical. To ensure broader access to these types of analyses to a wider range of laboratories, this approach uses 16S rRNA gene analyses that simplify the initial process. This protocol offers a practical and scalable solution for initial investigations and can be further completed by deeper analyses using shotgun metagenomics. This approach employs ESwab™ devices, which are already commonly used in hospitals [20]. Here, we applied this method on cirrhotic patients admitted to the medical intensive care unit (ICU) of our hospital and compared with a control group of compensated cirrhotic outpatients recruited in the hepatology unit.

## Methods

### Patient data sampling

**Fig. 1.**
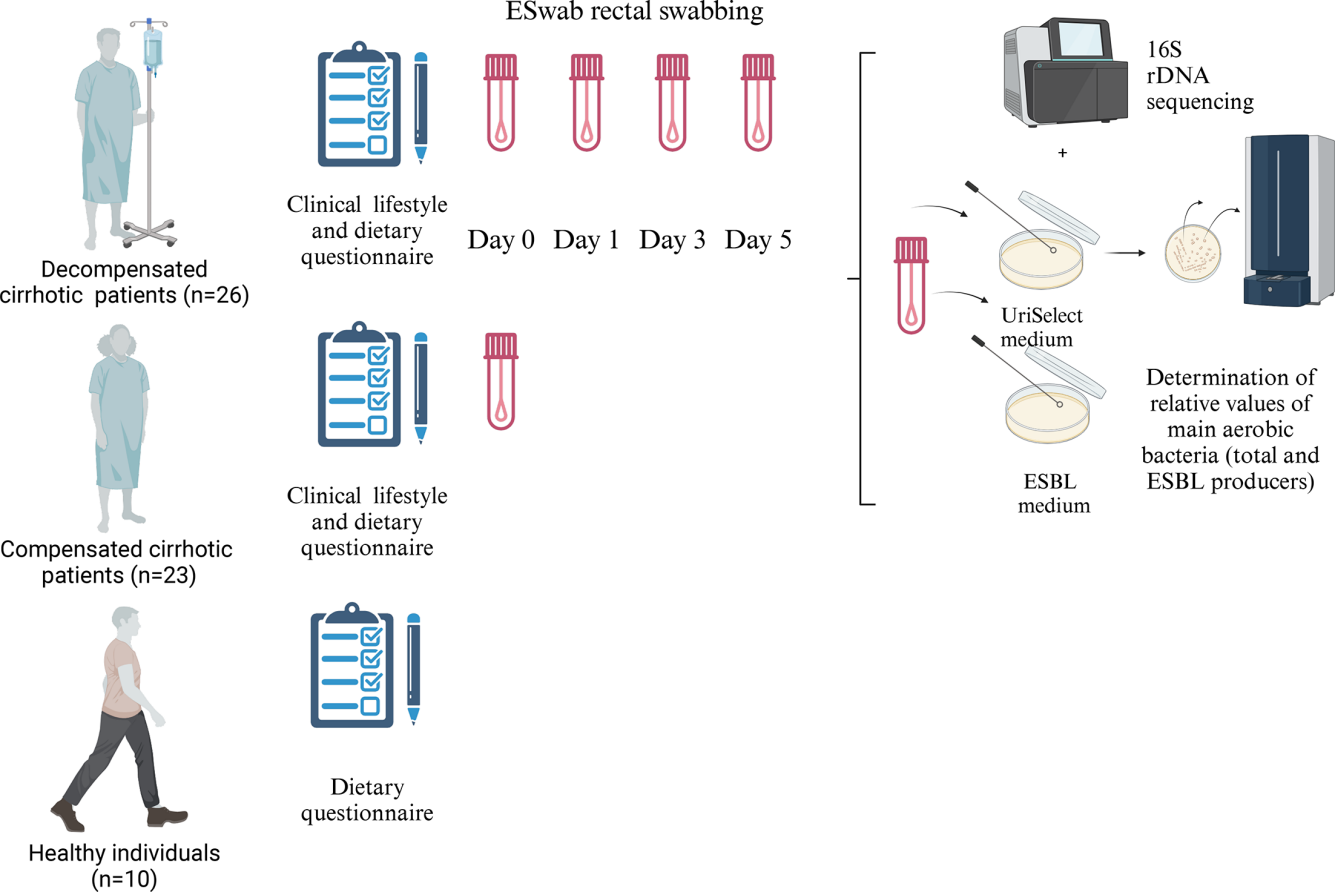
Diagrammatic overview of the sampling protocol for rectal swabs, including data collection processes (clinical, lifestyle and dietary questionnaire) for decompensated and compensated patients (*n*=26 and 23, respectively), alongside healthy volunteers (*n*=10) that undergo the dietary questionnaire. The different groups of patients and healthy volunteers are labelled. Each patient (from the decompensated and compensated groups) underwent rectal sampling using ESwab™ (Copan Diagnostics, Italy) device at day 0. Then, patients from the decompensated group underwent rectal swabbing at days 1, 3 and 5. Rectal ESwab™ are indicated in pink. For each patient, DNA extraction and 16S rRNA gene sequencing and diversity analysis in faecal microbiota were performed as previously described [19]. Aerobic microbiota quantification was performed as follows: for each sampling, 10 µl of the transport fluid of ESwab™ (Copan Diagnostics) was inoculated on UriSelect4 plates (Bio-Rad, La Coquette Marches, France) for semi-quantitative bacterial culture, for 18–24-h incubation at 37 °C without any supplemental in the atmosphere. After an overnight incubation, semi-quantification was performed using the abaque usually used for urine bacterial semi-quantification. Then, the relative quantification of each point was obtained by dividing the result of each aerobic bacteria on the total amount of all bacteria. The healthy volunteer’s data were only used to validate the diet score used in this study.

Patients enrolled in this study were hospitalized in the ICU and the hepatology department of the Jean Verdier Hospital in Bondy for cirrhosis decompensation between March 2017 and April 2018. A group of compensated cirrhotic outpatients regularly followed in the hepatology unit from the same hospital, at the Jean Verdier Hospital, served as a control group for the study (Fig. 1). The figure was designed using the BioRender website (www.biorender.com).

#### Lifestyle data

We prospectively collected lifestyle data including the body mass index (BMI), a diet score we developed in this study (SOM, available in the online Supplementary Material) and the EPICES score (Evaluation of Deprivation and Inequalities in Health Examination Centres) [21]. The calculation of BMI followed the conventional formula: dividing the individual’s body weight in kilogrammes by the square of their height in metres.

To compute the diet score, we developed an easy-to-use intensive care questionnaire including the major categories detailed in the guidelines of the French national health programme for nutritional scores [22] and adapted a score developed by Chaltiel *et al*. (SOM3 Table S1) [23]. With the exception of nuts, there was a corresponding category in our questionnaire for each dietary component of Chaltiel *et al*. [23]. In a few cases, we merged several of our categories (e.g. sugary foods). For two categories (red meat and processed meat), Chaltiel *et al*. relied on the quantity of food per week or day expressed in grammes or litres [23]. We considered a portion of red meat to weigh 175 g and a portion of processed meat to weigh 150 g. Similarly, we considered that a sweet-tasting beverage corresponded to 33 cl (a can) and that an alcoholic beverage corresponded to a glass of 20–25 g. The adapted scores are presented in the SOM3 Table S2. Finally, we used the following formula from Chaltiel *et al*. [23] to compute scores for each participant as the sum of each component *i* multiplied by its associated weight and divided by its maximum absolute value:

$$NutritionScore\;=\sum_{i}\left({component}_{i}\;\times \;\frac{{weight}_{i}}{\max\left(abs\left({component}_{i}\right)\right)}\right)$$

To validate this score, we also proposed this questionnaire to 11 healthy individuals (showing no signs of illness). We compared the diet scores among groups of individuals (healthy individuals and the two cirrhotic groups) and among cirrhosis types using the Wilcoxon test with Hochberg correction. Additionally, we fitted linear regressions to assess the association between patient status (or type of cirrhosis) and the diet score while controlling for confounding factors, age and sex.

The EPICES score, a measure of social deprivation, was calculated from an intricate evaluation of 11 distinct parameters. Each question carried its coefficient, with the total score incrementing with affirmative responses. As elucidated elsewhere [21], a higher EPICES score correlates with greater levels of deprivation experienced by the patient.

#### Clinical data

The clinical data describing the disease history and the current episode were delineated through a structured questionnaire encompassing various parameters (SOM). These included the classification of cirrhosis, the cause of hospital admission, factors essential for calculating the risk of extended spectrum beta-lactamase (ESBL)-producing *Enterobacteriaceae* colonization (RiskESBLScore), the occurrence of a sepsis episode during hospitalization and the description of antibiotic treatment during hospitalization. The RiskESBLScore (also introduced in this study) was calculated by summing the responses for distinct items. These items encompassed a history of MDRO infections in the last 3 months, antibiotic usage within the preceding 3 months, recent hospitalizations within the last 3 months, birth in a foreign country with high endemicity of MDRO colonization and recent travel in a foreign country within the past 3 months. We used a French recommendation document of experts in 2019 to treat MDRO and listed the ESBL risk factors after a large review of the literature [24]. Responses were dichotomously coded as absent (0) or present (1).

### Patient gut microbiota sampling

**Fig. 2.**
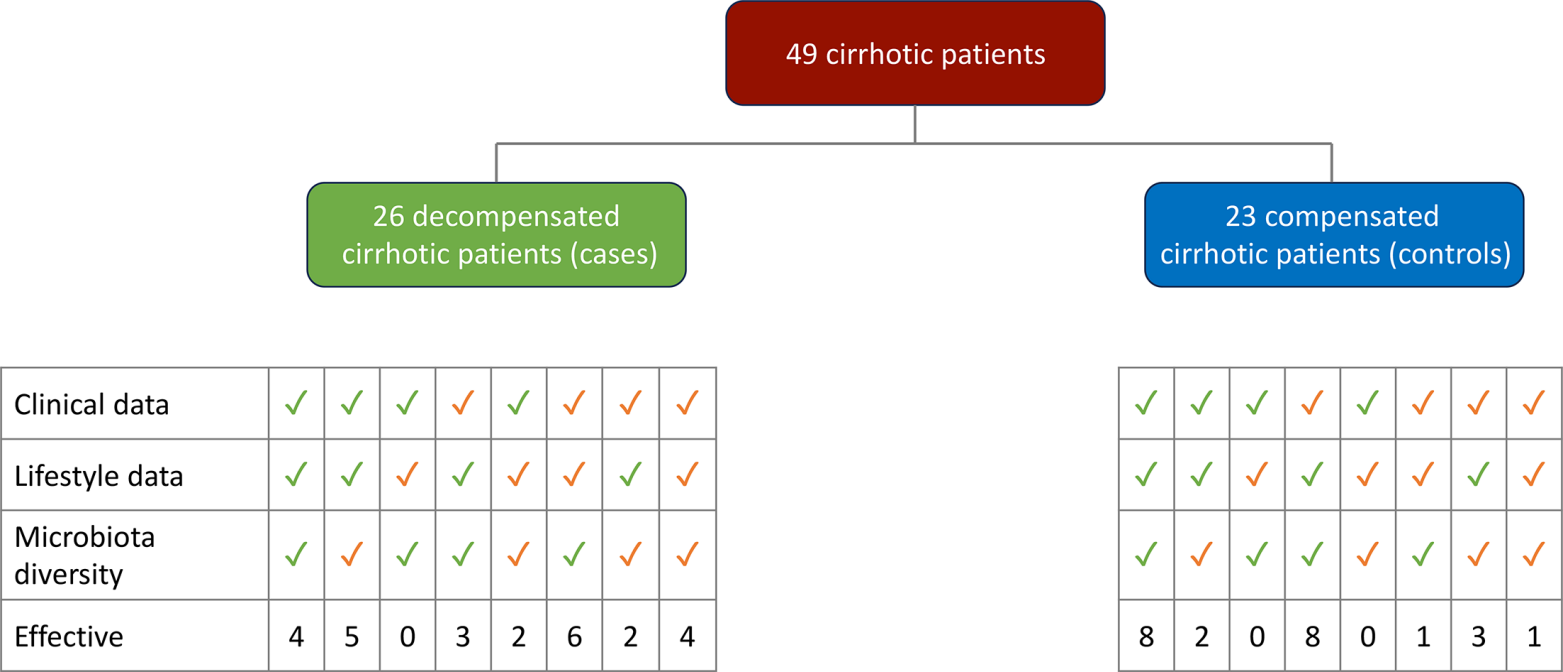
Description of the data collection of the two groups of compensated and decompensated cirrhotic patients. The completeness/incompleteness of clinical, lifestyle and microbiota data (aerobic and total) is indicated in green/orange.

Each patient (from the case and control groups) underwent rectal sampling using ESwab™ (Copan Diagnostics, Italy) device at day 0. Then, patients from the decompensated group underwent rectal swabbing at days 1, 3 and 5 (Fig. 2).

### Total microbiota sequencing and analysis

The DNA extraction and 16S rRNA gene sequencing and diversity analysis were performed as previously described [20].

### Aerobic microbiota quantification

For each sampling, 10 µl of the transport fluid of ESwab™ (Copan Diagnostics) was inoculated on UriSelect4 plates (Bio-Rad, Marnes La Coquette, France) for semi-quantitative bacterial culture, for 18–24-h incubation at 37 °C without any supplemental in the atmosphere. After an overnight incubation, semi-quantification was performed using the abaque usually used for urine bacterial semi-quantification. We classified each bacterium in four main groups to describe aerobic gut microbiota: *Escherichia coli*, *Enterococcus faecalis*, other Gram-negative bacteria and other Gram-positive bacteria. Then, the relative quantification of each point was obtained by dividing the result of each aerobic bacteria on the total amount of all bacteria.

### ESBL presence determination

ESBL-producing *Enterobacteriaceae* colonization was assessed through rectal swabs obtained from each patient. Subsequently, each swab was cultured on CHROMagar™ ESBL agar (CHROMagar, Saint-Denis, France) to facilitate the selection of Gram-negative bacteria, demonstrating resistance to third-generation cephalosporins. The resistance was confirmed using the B-lacta test (Bio-Rad, Marne La Coquette, France). Subsequently, species-level identification was carried out using matrix-assisted laser desorption/ionization-time of flight (MALDI-TOF) technique (Bruker, Champs-sur-Marne, France), a robust and reliable method widely used in medical laboratories. This technique provides scores, which are considered valid above 2.

### Power analysis

We conducted a power analysis to determine the probability of detecting an effect under our sample size constraints with an ANOVA (R package pwr) [25]. The sample effect corresponded to the Eta-squared estimate of the ANOVA, and the significance level was set to 0.05.

### Statistical analyses

All comparisons between groups were performed using nonparametric tests (Wilcoxon or Fisher exact tests). For multivariate analyses, data were divided into four categories corresponding to quartiles (0–25%, 25–50%, 50–75% and 75–100%). We performed a multiple correspondence analysis (‘MCA’ function in the R package FactoMineR [26]) to visualize the relationships between the variables. We determined the most characteristic categories according to each dimension with a factor analysis (‘dim desc’ function in the R package FactoMineR [26]). To statistically test the association among variables, we fitted a logistic regression with either clinical or lifestyle variables as predictors of the patient status (compensated or decompensated).

## Results

### Collecting data in ICUs is challenging

The study population comprised 49 cirrhotic patients, 26 individuals diagnosed with decompensated cirrhosis and 23 individuals with compensated cirrhosis. Among the decompensated cirrhotic patients, only 4 (15%) had a complete collection of both questionnaire data (clinical and lifestyle data) and rectal interpretable sample (total and aerobic microbiota diversities), 18 (69%) patients had incomplete data or encountered issues with either incomplete or unexploitable samples and 4 (15%) had both incomplete and unexploited samples. Among the compensated cirrhotic patients, 8 (35%) had a complete collection of both data (clinical and lifestyle data) and rectal interpretable sample (microbiota diversities) that was significantly higher compared to decompensated patients, 14 (61%) patients had incomplete data or encountered issues with either incomplete or unexploitable samples and only 1 (4%) had both incomplete and unexploited samples (SOM3 Tables S2, S7 and Fig. 2). The larger numbers of missing data were found for biological data of compensated patients and for MDRO antecedents prior antibiotherapies in both groups (SOM3 Table S7). Microbiota 16Sr RNA gene analysis could be performed for 50% and 26% of decompensated and compensated patient groups, respectively. None of the patients were excluded due to the fact that they all have at least data or samples to interpret.

### Evaluation of the diet score and global lifestyle characteristic observation reveals greater practice of sport and better diet for compensated cirrhotic patients

**Fig. 3.**
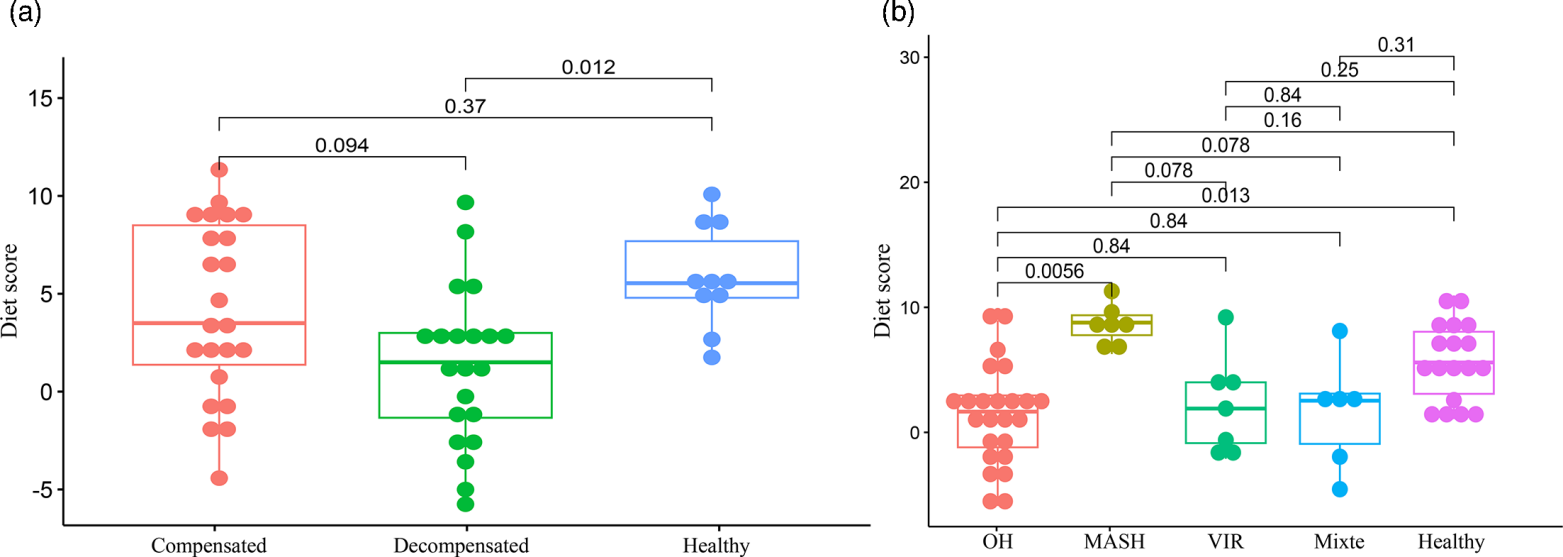
Evaluation of the diet score. We evaluated the distribution of the diet score for (a) compensated and decompensated cirrhotic patients and healthy volunteers and for (b) the different types of cirrhosis. We performed pairwise Wilcoxon tests with Hochberg correction for multiple tests.

First, to validate our diet score, we compared the diet score among patients (23 compensated and 21 decompensated for which we had nutrition data) and 11 healthy volunteers (SOM3 Table S4). We found significantly higher diet scores for healthy volunteers compared to decompensated patients (*P* value=2.30e−02 after the Benjamini–Hochberg correction) (Fig. 3). The other comparisons were not significant. However, when comparing only cirrhotic patients, we found a weak significant difference between compensated patients (median diet score=3.5) and decompensated patients (median diet score=1.5) (*P* value=4.70e−02) (Table 1). Similar results were obtained by fitting a linear model for the patient status in the function of the diet score, with significantly higher scores for healthy volunteers [effect size: 0.381 (0.124; 0.748), *P* value=1.34e−02] or compensated patients [effect size: 0.162 (0.019; 0.328), *P* value=3.66e−02] compared to decompensated patients (SOM3 Table S8). The associations were not significant anymore when including age and sex as confounding factors, probably because of the small sample sizes. However, we noted that the absolute values of the effect sizes for the interaction terms were smaller (<1.9 and <0.7 for healthy volunteers and compensated patients, respectively) than the effect size for diet score [effect sizes: 3.274 (−20.347; na) and 0.880 (−0.660; 5.746), respectively] (SOM3 Table S8).

**Table 1.** Clinical and lifestyle characteristics of decompensated and compensated cirrhotic patients

	Decompensated cirrhotic patients (*n*=26)	Compensated cirrhotic patients (*n*=23)	*P* value
**Demographic data**			
Age median (min–max)	59 (43–73)	61 (43–73)	0.95
Sex (F/M)	2/24	4/19	0.40
**Clinical characteristics**			
Cause of cirrhosis			**<1e−04**
Alcohol [*n* (%)]	20 (77)	5 (22)	**1.6−04**
MASH [*n* (%)]	1 (4)	6 (26)	**4.1e−02**
Viral origin [*n* (%)]	1 (4)	7 (30)	**1.9e−02**
Mixt [*n* (%)]	2 (8)	5 (22)	0.23
Other or na [*n* (%)]	2 (8)	0 (0)	0.49
Motif of admission			**<1e−04**
Day care control [*n* (%)]	0 (0)	23 (100)	**<1e−04**
Gastrointestinal haemorrhage [*n* (%)]	19 (73)	**0** (0)	**<1e−04**
Infection [*n* (%)]	4 (15)	0 (0)	0.11
Other or na [*n* (%)]	3 (12)	0 (0)	0.24
Other gravity indicators			0.11
Presence of ascites [*n* (%)]	10 (38)	0 (0)	**7.9e−04**
Presence of hepatic encephalopathy [*n* (%)]	6 (23)	0 (0)	**2.4e−02**
Presence of diabetes [*n* (%)]	10 (38)	4 (17)	0.13
Infectious events during hospitalization			1.00
Sepsis during hospitalization [*n* (%)]	2 (8)	0 (0)	0.49
Antibiotic during hospitalization [*n* (%)]	23 (88)	0 (0)	**<1e−04**
**Lifestyle characteristics**			
BMI median (min–max)	27.58 (19.05–37.65)	28.70 (20.06–39.89)	0.35
Diet score median (min–max)	1.5 (−5.75 to 9.67)	3.5 (−4.42 to 11.33)	**4.7e−02**
Sport score median (min–max)	0.5 (0–3)	3 (0–7)	**7.5e−03**
Precariousness score median (min–max)	46.74 (21.89–100)	42 (8.28–82.84)	0.30
**Microbiological characteristics**			
Shannon diversity median (min–max)	2.27 (0.10–2.66)	2.78 (0.83–3.34)	**7.2e−04**
ESBL presence [*n* (%)]	5 (19)	4 (17)	1.00
ESBL risk score median (min–max)	3 (1–5)	0.5 (0–3)	**2.1e−03**

We choose to present characteristics for which less than 20% of data were missing data per group (compensated or decompensated patients) except for Shannon diversity, sport, precariousness and ESBL risk scores for which 50% of decompensated patient data were missing data (SOM Table S7). Percentages represent the proportions of the positive characteristic on the number of available data per group. We performed Fisher’s exact test for categorical data and Student’s t-test or Welch’s t-test for quantitative data with equal or unequal variances respectively. NA : non applicable.

Next, we compared the diet score among cirrhosis types (Fig. 3). We found a higher diet score for metabolic dysfunction-associated steatohepatitis (MASH) patients compared to alcohol-associated cirrhosis (OH) patients (*P* value=5.60e−03). The other comparisons were not significant among cirrhosis types, probably because of the small sample sizes. Indeed, given our limited sample size, the power analyses indicated that the probability of detecting differences in the diet score was particularly low among volunteers, compensated and decompensated patients (0.18) and slightly larger among types of cirrhosis (0.62).

Decompensated patients had a higher median sport score [3] compared to compensated patients (0.5) (*P* value=7.51e−03) (Table 1). However, there was no significant difference in the precariousness score between the two groups (*P* value=0.30).

### Different clinical picture among decompensated and compensated cirrhotic patients

Decompensated cirrhotic patients exhibited significant differences in the type of cirrhosis compared to compensated cirrhotic patients (*P* value<1.00e−04) (Table 1). The majority of decompensated cirrhotic patients had alcohol-related cirrhosis (83%), whereas compensated cirrhotic patients predominantly had cirrhosis of MASH origin (26%). Gastrointestinal haemorrhage was the most common reason for hospital admission among decompensated cirrhotic patients (73%), while compensated cirrhotic patients were primarily admitted for day care control (100%) (*P* value <1.00e−04). BMI did not significantly differ between the two groups (*P* value=0.35). Sepsis during hospitalization was observed in two decompensated cirrhotic patients, whereas none of the compensated patients developed sepsis (*P* value=0.49). Antibiotic treatment during hospitalization was significantly more common among decompensated cirrhotic patients compared to compensated patients (*P* value<1.00e−04).

### Richer microbiota for compensated cirrhotic patients

Microbiological analysis revealed differences in the microbial diversity between decompensated and compensated cirrhotic patients. We compared the alpha diversity (Shannon index) between decompensated cirrhotic (*n*=13) and compensated cirrhotic patients (*n*=17) (SOM Fig. S1). By fitting a logistic regression, we found a significant negative association between decompensated cirrhotic patients and microbiota diversity, for both operational taxonomic unit (OTU) richness [effect size: −0.06 (−0.12; −0.01), *P* value=0.026] and Shannon index [effect size: −1.41 (−2.82; −0.45), *P* value=0.015] (SOM Table S9). To evaluate the effect of confounding factors, here age and sex, we added the interaction terms between these factors and microbiota diversity in the logistic regression (SOM Table S9). The association was not significant anymore; however, the absolute values of the effect sizes of OTU richness [effect size: −0.09 (−0.23; 0.03)] and Shannon index [effect size: −2.16 (−6.65; 1.03)] were much bigger than the effect sizes of the interaction terms (effect sizes<0.01 for OTU richness and <0.21 for Shannon index) (SOM Table S9). These results suggested that while not significant, probably because of our very limited sample size, there is a difference in microbiota diversity between compensated and decompensated patients and a negligible effect of age and sex.

Concerning the ESBL-producing bacterial presence, we initially selected bacteria resistant to third-generation cephalosporins using ESBL agar, which contains a third-generation cephalosporin. The resistance was confirmed using the B-lacta test. Subsequently, species-level identification was carried out using the MALDI-TOF technique, a robust and reliable method widely used in medical laboratories. In our study, all bacteria identified as resistant to third-generation cephalosporins were classified as *Escherichia coli*. Given the epidemiology, we considered those third-generation cephalosporin-resistant *Escherichia coli* as ESBL producers. The prevalence of ESBL presence did not significantly differ between decompensated and compensated patients (*P* value=1.00) (Table 1). However, the ESBL risk score showed a significant difference between the two groups, with compensated patients having a lower median score (0.5) compared to decompensated patients [3] (*P* value=2.14e−03).

These findings highlight notable distinctions in clinical, lifestyle and microbiological characteristics between decompensated and compensated cirrhotic patients, underscoring the complexity of cirrhosis and its associated factors.

### Multivariate analysis

**Fig. 4.**
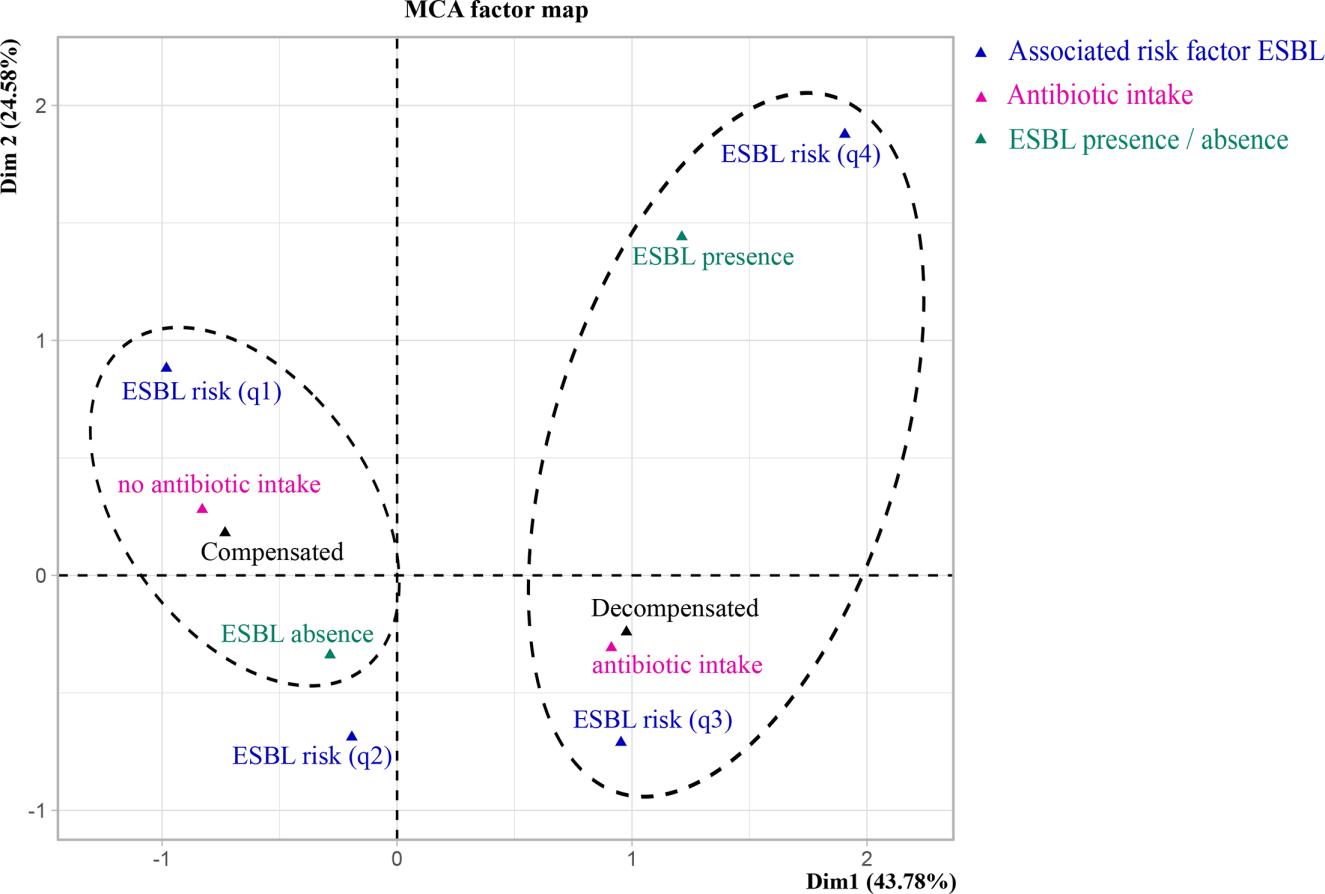
MCA of clinical variables (associated risk factor ESBL, antibiotic intake and presence/absence of ESBL) for 12 compensated and 9 decompensated patients (patients without missing values). The most characteristic categories according to dimension 1 are encircled. The associated risk factor ESBL scores indicate a low risk of ESBL carriage [ESBLRisk(q1)] to a high risk of ESBL carriage [ESBLRisk(q4)].

**Fig. 5.**
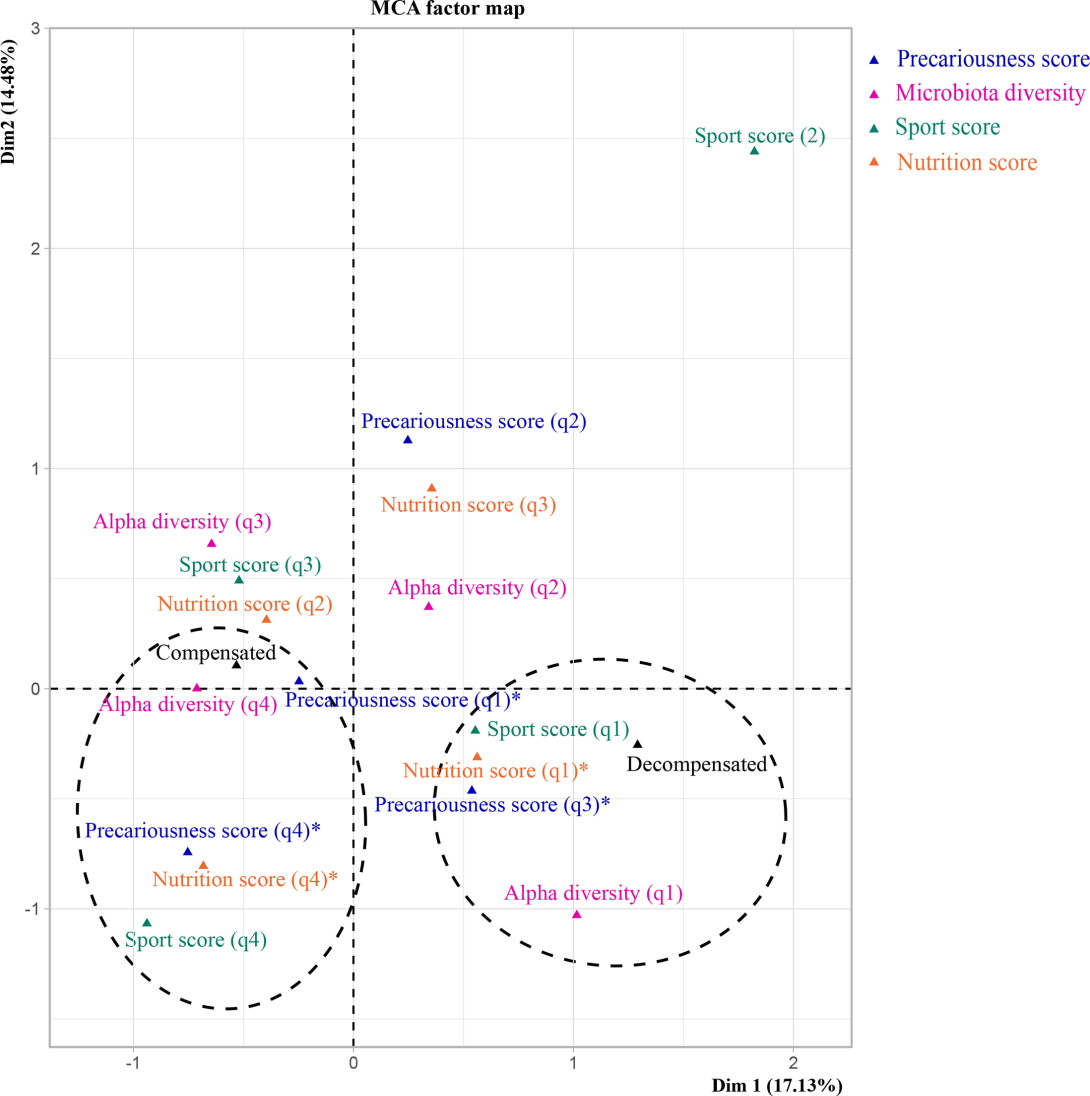
MCA of lifestyle variables and microbiota diversity (precariousness score, sport score and diet score) for 17 compensated and 7 decompensated individuals (individuals without missing values). The most characteristic categories according to dimension 1 are encircled. The precariousness scores indicate a low precarious situation [PrecariousnessScore(q1)] to a great precarious situation [PrecariousnessScore(q’)]. The diet scores indicate a healthy diet [NutScore(q1)] to an unbalanced diet [NutScore(q4)]. The sport scores indicate little practice [SportScore(q1)] to an important practice [SportScore(q4)]. The microbiota diversity scores indicate a low diversity [AlphaDiversity(q1)] to a great diversity [AlphaDiversity(q4)]. The asterisk (*) means not characteristic categories according to dimension 1.

We evaluated the relationships among four clinical variables (associated risk factor ESBL, antibiotic intake and presence/absence of ESBL) and among three lifestyle variables (precariousness score, sport score and diet score) and microbiota diversity (SOM Tables S4–S6). The ordination plot suggested an association between patient status (compensated or decompensated patients) and the three clinical variables, antibiotic intake, presence or absence of ESBL and associated risk factor ESBL scores (Fig. 4). We corroborated these results for two variables, antibiotic intake and associated risk factor ESBL scores. We found a negative association between decompensated patient status and no antibiotic intake [effect size: −3.69 (−6.93; −1.45), *P* value=0.005] and a positive association between decompensated patient status and associated risk factor ESBL scores [effect size: 1.99 (0.68; 4.80), *P* value=0.034] by fitting a logistic regression (SOM Table S10). Regarding lifestyle variables and microbiota diversity, the ordination plot suggested an association between patient status and two variables, sport scores and microbiota diversity (Fig. 5). The association between decompensated patient status and lower microbiota diversity was further supported by a logistic regression [effect size: −1.53 (−3.01; −0.49), *P* value=0.012] (SOM Table S10).

### Concomitance of *Escherichia coli* ESBL frequency increase and sepsis in decompensated cirrhotic patients

**Fig. 6.**
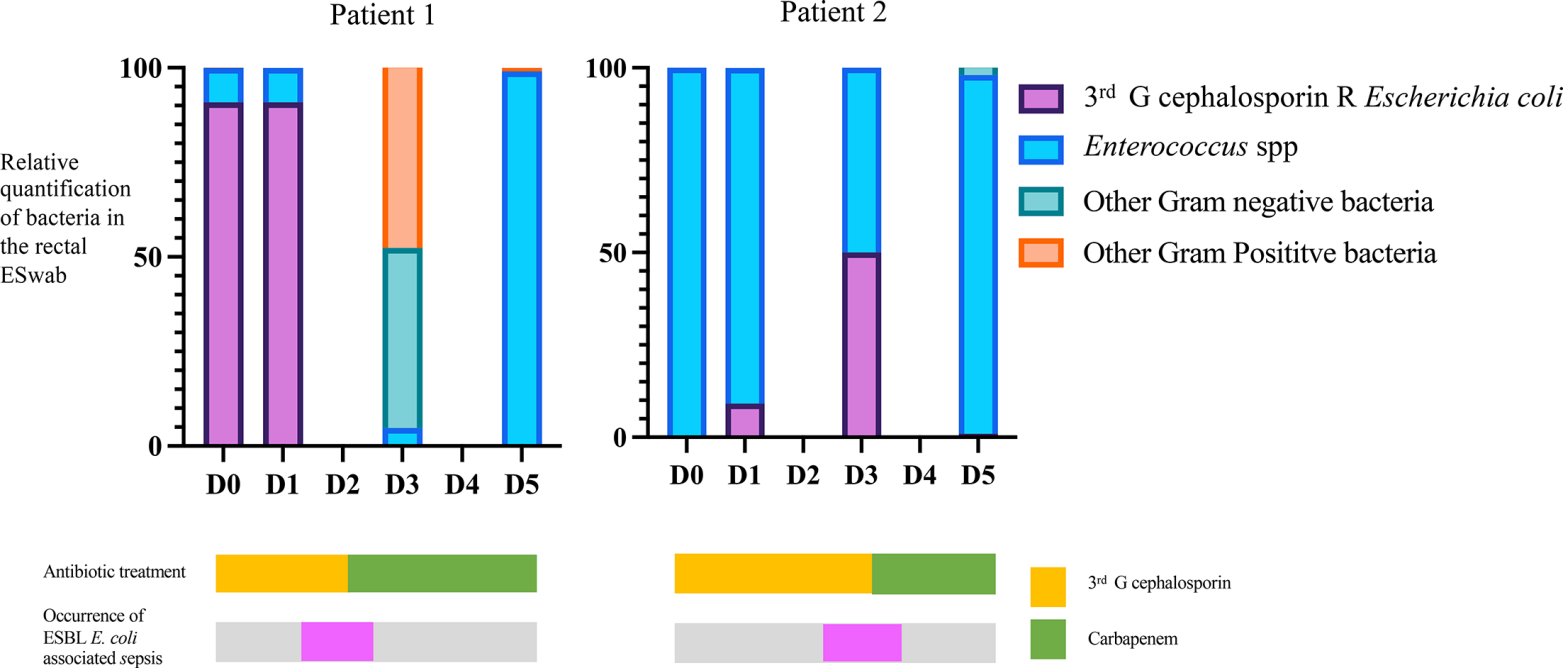
Temporal dynamics of bacterial relative frequencies in two sepsis patients during.

We focused then on relative the quantification of aerobic microbiota using the transport fluid of ESwab™ directly inoculated on UriSelect4 and on ESBL media. This gave us on one hand the relative quantification for each sample of the main groups of bacteria (*Escherichia coli*, Enterococcus sp. and other Gram-negative and Gram-positive bacteria, we called aerobic gut microbiota) and the presence/absence of third-generation cephalosporin Gram-negative bacteria on the other hand. We analysed relative semi-quantitative aerobic microbiota data from all patients at day 0 (D0), comparing decompensated and compensated patients but found no discernible difference. Additionally, we examined data between D0 and D5, focusing on patients who developed sepsis during hospitalization (Fig. 6). These patients had initially received non-active antibiotics against ESBL producers upon admission but later developed sepsis caused by ESBL-producing *Escherichia coli*. A retrospective analysis of the temporal dynamics of the relative quantification of aerobic gut microbiota and determination of colonization of ESBL Gram-negative bacteria was carried out for the two patients at D0, D1, D3 and D5. The two patients presented ESBL-producing *Escherichia coli* bacteraemia on day 1 and day 2, respectively, and were taking third-generation cephalosporin. Besides, both patients showed an increase in the quantification of ESBL-producing *Escherichia coli* prior to the bacteraemia (Fig. 6).

## Discussion

In this study, we aimed to propose a pragmatic approach combining rectal swabbing and simple questionnaires applicable in difficult units (ICU or emergency units) treating particularly fragile patients such as immunocompromised patients. In those units, obtaining whole stool samples is often impractical before antibiotherapy for incoming patients. On the contrary, rectal swabbing is already integrated into paramedical routine care for hygiene analyses. Besides, conducting clinical research with very detailed and time-consuming questionnaires can be complicated in departments where staff are often stretched to the limit. We employed this methodology on a cohort of cirrhotic patients, contrasting decompensated cirrhotic individuals in ICUs with compensated cirrhotic individuals.

The study included 49 cirrhotic patients, 26 with decompensated cirrhosis and 23 with compensated cirrhosis. Despite efforts to streamline data and sample collection, variability in completeness highlighted the challenges in clinical data and sample collection with patients hospitalized in the ICU. For example, it is absolutely necessary to remind medical and nurses’ staff to obtain rectal swabs with sufficient faecal materials; indeed, it was often insufficient leading to 50% missing data for the microbial diversity index in decompensated patients (SOM 3 Table S7). Moreover, it is important to note that for patients who promptly received antibiotics upon admission, whole stool analysis would have been compromised. Indeed, samples would have been collected post-antibiotic treatment, rendering them unsuitable for analysis.

The probability of missing data is considerable, due to the intrinsic difficulties in acquiring comprehensive clinical information and high-quality samples in ICU and emergency settings, as demonstrated in this study. This highlights the necessity of including a larger number of patients to ensure robust results. This complexity suggests the need for further studies to obtain more interpretable clinical results. However, thanks to our simplified protocol including faecal material sampling and lifestyle/clinical questionnaires, we were able to analyse data for all 49 patients that presented at least some clinical and lifestyle data and for 30 patients with clinical or lifestyle data and exploitable samples of rectal swabs (SOM3 Tables S2 and S7).

Decompensated patients primarily had alcohol-related cirrhosis and were frequently hospitalized for gastrointestinal haemorrhages. In contrast, compensated patients often had MASH-related cirrhosis and were admitted for routine care. Decompensated patients had significantly higher antibiotic use, indicating more severe health conditions. They also exhibited higher nutrition and sport scores, reflecting lifestyle adjustments to manage their condition. No significant difference was found in the precariousness score between the groups. Microbiologically, compensated patients had higher microbial diversity (OTU richness and Shannon index) than decompensated patients. The lower ESBL risk score in decompensated patients corresponded to higher antibiotic exposure, impacting microbial diversity. Shao *et al.* already observed lower diversity in decompensated patients, validating part of our observations [27]. Our multivariate analysis revealed that decompensation was associated with higher antibiotic intake and ESBL risk scores, while compensated patients showed the opposite trends. Decompensated patients also had lower microbial diversity, highlighting the interplay between clinical severity and microbiota composition in cirrhosis management. Numerous studies exist that try to decipher risk factors of decompensation and the appearance of bacterial infections associated with gravity [17,28, 29] However, to our knowledge, none have evaluated all these characteristics together (decompensated vs compensated cirrhosis, ESBL-producing bacterial presence, risk factors and microbial diversities).

Finally, the study of bacterial population dynamics in sepsis patients revealed an increase in the *Escherichia coli* relative frequency, coinciding with the emergence of ESBL-producing *Escherichia coli*. We were able to analyse complete follow-ups of five other decompensated cirrhotic patients. However, only one exhibited an invasion by a bacterium from the *Enterococcaceae* family, and this patient showed no signs of sepsis (data not shown). Further prospective studies, with larger cohorts, are necessary to corroborate this result. Nevertheless, we suggest incorporating this follow-up data into antibiotic selection for managing sepsis. Prado *et al.* observed a correlation between MDRO presence and an increase in bacterium associated with infections in cirrhotic patients using qualitative data [29]. We think that more fine quantitative results could give rigorous predicting factors. This finding emphasizes the need for timely and appropriate antibiotic treatment to manage and prevent sepsis effectively. In this study, we offered a simple way to help the management of antibiotic therapy by proposing new follow-up of severe patients by aerobic gut and antibiotic-resistant Gram-negative bacterial relative quantifications.

Given the limited number of patients but the promising results, this toolbox shows potential use for larger prospective studies in order to provide new insights into the severity of cirrhosis. It also identifies predictive characteristics that could facilitate managing cirrhosis and infectious diseases in cirrhotic patients. Moreover, this method could be applied to other numerous pathologies where dysbiosis or immune responses are linked to disease management outcomes.

## Conclusion

In this study, we present a streamlined approach for assessing total diversity and aerobic microbiota using rectal swabs, along with simplified demographic, lifestyle (including dietary habits) and clinical recording methods. These techniques are not only applicable in ICUs but also in clinical laboratory settings. They can be significantly developed to conduct clinical research in emergency and ICUs, where sampling clinical and biological data can be challenging. Additionally, these methods are beneficial for patients with diseases associated with microbiota disorders or those at high risk of opportunistic pathogens originating from the gut.

## supplementary material

10.1099/jmm.0.001964 Uncited Supplementary Material 1.

## Abbreviations

BMI: body mass index
ICU: intensive care unit
MALDI-TOF: matrix-assisted laser desorption/ionization-time of flight
MASH: metabolic dysfunction-associated steatohepatitis
MCA: multiple correspondence analysis
MDRO: multidrug-resistant organism
