# Supplementary material for: Comprehensive toolkit integrating lifestyle and clinical questionnaires with gut microbiota profiling via rectal swabs: application in intensive care cirrhotic patients
Source: J Med Microbiol. 2025 Mar 6;74(3):001964. doi: 10.1099/jmm.0.001964 (PMC11936375; doi:10.1099/jmm.0.001964)
Supplement: Uncited Supplementary Material 1. [file jmm-74-01964-s001.pdf]

Questionnaire

Demographic Data

Gender: F M
Age:
Weight
Height
Occupation:

Socio-Economic Questionnaire (Precariousness)

Number of children
Type of social insurance
Secu CMU PUMA
AME
Private insurance

Type of supplementary health insurance
Private mutual insurance
Complementary CMU / PUMA

EPICE Score
Do you sometimes meet with a social worker? YES NO
Do you have supplementary health insurance? YES NO
Do you live with a partner? YES NO
Are you a homeowner? YES NO
Do you experience periods in the month where you face real financial difficulties in meeting basic needs (food, rent, electricity...)? YES NO
Have you engaged in sports in the past 12 months?
Have you attended any shows in the past 12 months? YES NO
Have you been on vacation in the past 12 months? YES NO
In the last 6 months, have you had contact with family members other than your parents/children? YES NO
In case of difficulties, do you have people in your circle who can provide you with accommodation or material assistance? YES NO

Physical Activity

Sport
Do you consider yourself very active?
Regular exercise >30 minutes/day or not
30 minutes of brisk walking per day? YES NO
Weekly exercise? YES NO
Occasional exercise? YES NO

Diet questionnaire

In the last month, how often did you eat or drink the following foods/beverages?

	**Never**	**1 - 3 times/ month**	**1- 2 times / week**	**3- 5 times / week**	**Once per day**	**>2 times / day Precise how many**
Red meat (beef, lamb, mutton, pork…)						
Poultry and white meats (chicken, rabbit…)						
Processed meats (including white ham)						
Fish and seafood						
Eggs						
Fruits and vegetables (including fruit or vegetable juices)						
Starches in general (Pasta, rice, bread, legumes, potatoes…)						
Whole foods: whole grain bread, pasta or rice						
Legumes (lentils, peas, chickpeas, etc.)						
Yogurts						
Cheese						
Milk						
Breakfast cereals						
Non-fat sugary products (Candies, honey, jam…)						
Fatty-sweet products (chocolate, ice cream, pastries, baked goods, biscuits, sweet dairy desserts…)						
Fatty-salty products (Savory snacks, hamburgers, pizza, fries, etc.)						
Fats (Butter, margarine, vegetable oil…)						

Sugary drinks (non-diet)						
Red wine, white wine						
Beer						
Aperitifs and digestifs						

Reason for Patient Admission

Circle the exact answer(s):

Infection (to be considered as the main cause if there is no bleeding)
Ascites fluid infection
Other:
Gastrointestinal bleeding
Hepatic encephalopathy
Others:

Clinical Data concerning the Patient upon Admission to ICU

Cause of cirrhosis
Alcohol: YES NO
HBV/HCV: YES NO
NASH: YES NO
Other: (Specify):

Assessment of Current Decompensation
Evaluation of portal hypertension: (circle the exact answer)
Absent ascites:
Moderate ascites:
Abundant ascites:
Presence of hepatic encephalopathy YES NO
If yes, grade:
Gastrointestinal bleeding YES NO
Renal function evaluation
Renal failure YES NO
Liver function:
Current PT
Baseline PT

Comorbidities

Renal function: (circle the exact answer)
Normal
Renal failure: 1 2 3 4 5

Diabetes: (circle the exact answer)
Absent
Type 1
Type 2
Non-insulin requiring
Insulin requiring

Immunosuppression: (circle the exact answer)
Absent
HIV/AIDS stage (< 200 CD4)
Progressive solid cancer
Malignant hematopathy
Organ or bone marrow transplantation
Neutropenia <500
Congenital immunodeficiency
Immunosuppressive therapy (corticosteroids, chemotherapy, immunomodulatory treatment)

Sepsis after admission Yes/No

Date of diagnosis
Microbiological documentation YES NO
Entry point and type (describe):

Risk factors for MDRO /Histoty of antibiotic treatment

History of MDR infection in the last 6 months YES NO do not know

Antibiotic treatment in the last 6 months YES NO
Antibiotic #1:
Name:
Start date
Reason:

Antibiotic #2:
Name:
Start date
Reason:

Antibiotic #3:
Name:
Start date
Reason:

Long-term norfloxacin prophylaxis YES NO

Hospitalization in the previous year: YES NO
Patient born in France YES NO
If no, Country of birth:
In France since:

Before hospitalization, was the patient:
At home YES NO
Already hospitalized: YES NO

Travel in the last 3 months YES NO
If yes, return date
Country
Taking antibiotics during the trip YES NO
Diarrhea during the trip YES NO
Contact with a healthcare facility during the trip YES NO

Antibiotic treatment initiated during the hospitalization

Antibiotic (ATB) No. 1:
Name:
Initiation date
Time and date of first injection
Duration
Dosage
Reason:

ATB No. 2:
Name:
Initiation date
Time and date of first injection
Duration
Dosage
Reason:

ATB No. 3:
Name:
Initiation date
Time of first injection
Dosage
Reason:

Outcome

Date of discharge from intensive care (Circle the exact answer)
Alive discharge
Return home
Transfer to another department:

Deceased discharge

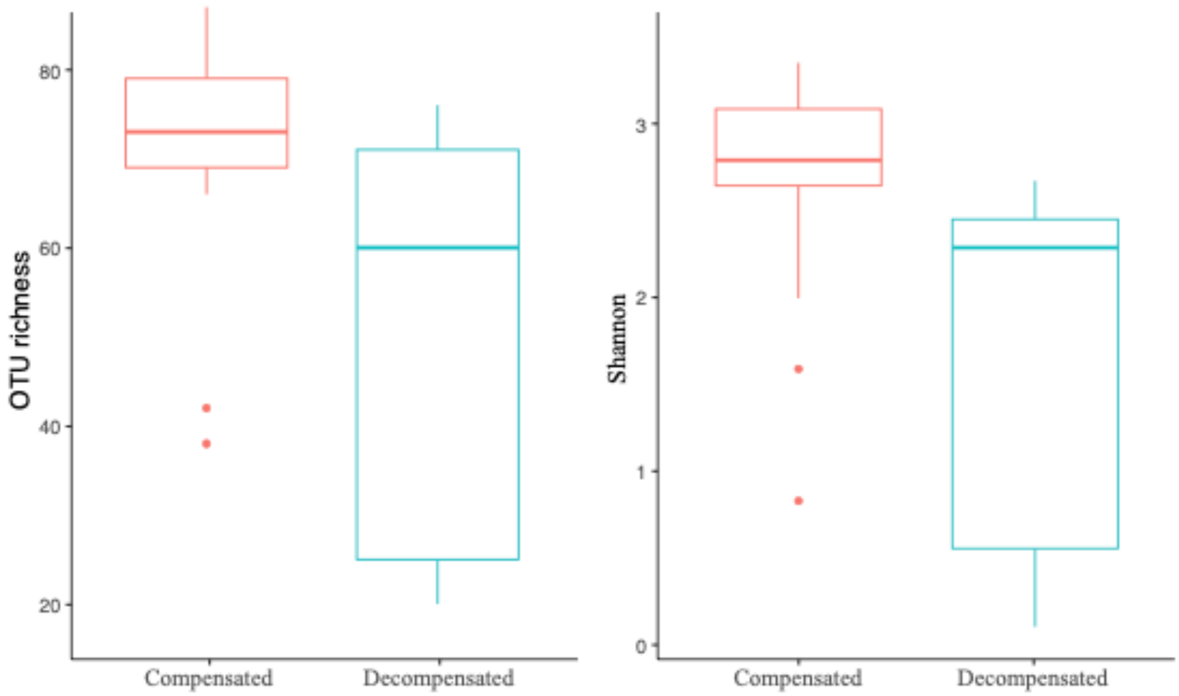

Figure S1. Evaluation of the microbiota diversity. We compared the microbiota diversity (OTU richness and Shannon index) among 17 compensated and 13 decompensated patients for which we had microbiota data.

Table S1. Correspondances between our nutrition score and the score developed by Chaltiel et al. 2019.

Category - our questionnaire	Category - Chaltiel et al. 2019	Weight	Scores					
			Never	1 to 3 times / month	1 to 2 times / week	3 to 5 times / week	1 / day	> 2 times / day
Viandes rouges	Red meat	2	0	0	0	-1	-2	-2
Charcuteries	Processed meat	3	0	0	-1	-2	-2	-2
Poissons et produits de la mer	Fish and seafood	2	0	0	1	0	0	0
Fruits et légumes	Fruits and vegetables	3	0	0	0	0	0	1
Aliments « complets »	Whole-grain food	2	0	0.5	0.5	0.5	1	1.5
Légumes secs	Legumes	1	0	0.5	0.5	1	1	1
Yaourts / Lait	Milk and dairy products	1	0	0	0	0.5	0.5	1
Produits sucrés non gras / Produits gras sucrés	Sugary foods	3	0	0	0	-1	-1	-2
Boissons sucrées (non light)	Sweet-tasting beverages	3	0	-0.5	-0.5	-0.5	-1	-2
Produits gras-salés	Salt	3	1	1	1	1	1	-1
Matières grasses	Added fat	2	1.5	1.5	1.5	1.5	1.5	0
Vin rouge, blanc / Bières / Apéritifs et digestifs	Alcoholic beverages	3	0	0	0	0	-1.5	-2

Table S2. Recapitulative table of patients and of their clinical and lifestyle data

PatientNumber	CaseControl	Age	Sex	DietScore	SportScore
Pat1	Control	70	M	2.17	7
Pat2	Control	65	M	6.33	1
Pat3	Case	58	M	9.67	1
Pat4	Control	64	M	11.33	1
Pat5	Control	64	M	7.50	0
Pat6	Case	62	M	-2.33	1
Pat7	Control	67	M	-1.00	3
Pat8	Control	54	M	6.67	0
Pat9	Control	53	M	8.83	7
Pat10	Control	73	F	3.25	3
Pat11	Case	63	M	5.17	0
Pat12	Case	51	M	-2.83	1
Pat13	Control	63	M	2.00	1
Pat14	Control	43	M	-2.00	7
Pat15	Case	60	F	NA	NA
Pat16	Control	51	F	9.00	5
Pat17	Control	59	F	8.17	3
Pat18	Control	57	M	9.67	3
Pat19	Case	67	M	3.00	2
Pat20	Case	66	M	2.75	NA
Pat21	Case	43	M	3.00	0
Pat22	Control	56	F	-0.50	5
Pat23	Control	70	M	9.17	4
Pat24	Control	61	M	2.00	3
Pat25	Case	70	M	3.00	NA
Pat26	Control	50	M	2.25	0
Pat27	Control	67	M	9.25	3
Pat28	Case	70	M	1.50	NA
Pat29	Case	58	M	5.58	0
Pat30	Case	51	M	3.17	NA

PatientNumber	CaseControl	Age	Sex	DietScore	SportScore
Pat31	Control	50	M	4.67	3
Pat32	Case	71	M	8.17	0
Pat33	Case	57	M	0.83	2
Pat34	Case	52	M	-0.25	3
Pat35	Case	61	M	2.50	0
Pat36	Case	71	M	NA	NA
Pat37	Case	52	M	NA	NA
Pat38	Case	69	M	-3.58	0
Pat39	Control	56	M	0.75	1
Pat40	Case	73	M	-1.33	0
Pat41	Case	NA	F	1.00	NA
Pat42	Control	61	M	-4.42	3
Pat43	Case	58	M	NA	NA
Pat44	Control	59	M	-1.83	0
Pat45	Case	46	M	-5.00	NA
Pat46	Case	58	M	-1.00	3
Pat47	Control	61	M	3.50	1
Pat48	Case	46	M	NA	NA
Pat49	Case	NA	M	-5.75	NA

PatientNumber	PrecariousnessScore	BodyMassIndex	ReasonAdmssion	CirrhosisType	AscitePresnce
Pat1	33.14	28.70	Day Hospitalization	Alcohol	0
Pat2	14.79	32.60	Day Hospitalization	NASH	0
Pat3	21.89	22.72	Infection	Alcohol	0
Pat4	20.11	35.83	Day Hospitalization	NASH	0
Pat5	55.03	35.83	Day Hospitalization	NASH	0
Pat6	46.74	19.05	Gastrointestinal	Alcohol	0
Pat7	55.03	25.80	Day Hospitalization	VIR	0
Pat8	50.29	23.88	Day Hospitalization	Alcohol	0
Pat9	68.04	35.93	Day Hospitalization	NASH	0
Pat10	27.21	NA	Day Hospitalization	Mixed	0
Pat11	57.99	26.08	Gastrointestinal	Alcohol	1
Pat12	50.29	27.17	Other	Alcohol	2
Pat13	56.8	25.56	Day Hospitalization	VIR	0
Pat14	8.28	25.14	Day Hospitalization	VIR	0
Pat15	NA	37.65	Gastrointestinal	Mixed	0
Pat16	82.84	23.18	Day Hospitalization	Alcohol	0
Pat17	81.66	33.59	Day Hospitalization	Mixed	0
Pat18	40.82	33.56	Day Hospitalization	NASH	0
Pat19	33.72	29.06	Gastrointestinal	Alcohol	0
Pat20	NA	31.05	Gastrointestinal	Alcohol	1
Pat21	91.72	33.06	Infection	Alcohol	2
Pat22	44.97	23.44	Day Hospitalization	VIR	0
Pat23	58.58	NA	Day Hospitalization	NASH	0
Pat24	40.82	25.91	Day Hospitalization	Alcohol	0
Pat25	NA	27.58	Gastrointestinal	Mixed	0
Pat26	42	29.41	Day Hospitalization	Mixed	0
Pat27	20.11	28.98	Day Hospitalization	VIR	0
Pat28	NA	NA	Gastrointestinal	Alcohol	1
Pat29	46.75	25.31	Gastrointestinal	Alcohol	0
Pat30	67.45	27.18	Gastrointestinal	Alcohol	0

PatientNumber	PrecariousnessScore	BodyMassIndex	ReasonAdmssion	CirrhosisType	AscitePresnce
Pat31	48.52	25.47	Day Hospitalization	VIR	0
Pat32	59.17	32.18	Gastrointestinal	NASH	2
Pat33	22.48	30.80	Gastrointestinal	Alcohol	0
Pat34	44.97	24.06	Gastrointestinal	Alcohol	0
Pat35	100	19.25	Gastrointestinal	Alcohol	0
Pat36	NA	NA	NA	NA	NA
Pat37	NA	NA	Gastrointestinal	VIR	0
Pat38	34.32	19.72	Infection	Alcohol	1
Pat39	36.69	32.87	Day Hospitalization	Alcohol	0
Pat40	43.79	30.30	Gastrointestinal	Alcohol	1
Pat41	NA	28.48	Gastrointestinal	Alcohol	0
Pat42	34.9	20.06	Day Hospitalization	Mixed	0
Pat43	NA	27.76	INFECTION	Alcohol	2
Pat44	43.79	39.89	Other	Mixed	0
Pat45	60.36	21.26	Gastrointestinal	Alcohol	0
Pat46	30.17	29.76	Gastrointestinal	Alcohol	0
Pat47	33.72	25.48	Day Hospitalization	VIR	0
Pat48	NA	NA	NA	NA	NA
Pat49	NA	NA	Alcohol	1	0

PatientNumber	HepaticEncephalpathy	GastrointestinalHaemorrhage	LowProthrombinRate	Diabete
Pat1	0	0	NA	0
Pat2	0	0	NA	1
Pat3	1	0	0	0
Pat4	0	0	NA	1
Pat5	0	0	NA	1
Pat6	0	1	0	0
Pat7	0	0	NA	0
Pat8	0	0	NA	0
Pat9	0	0	NA	NA
Pat10	0	0	NA	NA
Pat11	0	1	0	1
Pat12	1	0	1	1
Pat13	0	0	NA	0
Pat14	0	0	NA	0
Pat15	0	1	0	1
Pat16	0	0	NA	0
Pat17	0	0	NA	0
Pat18	0	0	NA	0
Pat19	1	1	0	0
Pat20	0	1	0	0
Pat21	0	1	1	0
Pat22	0	0	NA	0
Pat23	0	0	NA	1
Pat24	0	0	NA	NA
Pat25	0	1	0	1
Pat26	0	0	NA	0
Pat27	0	0	NA	NA
Pat28	0	1	0	0
Pat29	1	1	0	1
Pat30	0	1	0	1

PatientNumber	HepaticEncephalpathy	GastrointestinalHaemorrhage	LowProthrombinRate	Diabete
Pat31	0	0	NA	0
Pat32	0	1	1	1
Pat33	0	1	1	0
Pat34	0	1	0	1
Pat35	0	1	1	0
Pat36	NA	NA	NA	NA
Pat37	0	1	0	0
Pat38	1	0	0	0
Pat39	0	0	NA	0
Pat40	0	1	NA	1
Pat41	0	1	1	1
Pat42	0	0	NA	0
Pat43	1	0	1	0
Pat44	0	0	0	0
Pat45	0	1	NA	0
Pat46	0	1	0	0
Pat47	0	0	NA	0
Pat48	NA	NA	NA	NA
Pat49	1	0	0	NA

PatientNumber	AssociatedRiskFactorESBL	MultiDrugResistanceOrganismAntecedents	AntibioticTreatment6Months	NorfloxacineProphylaxis
Pat1	2	0	0	0
Pat2	0	0	0	0
Pat3	NA	NA	1	0
Pat4	0	0	0	0
Pat5	NA	NA	0	NA
Pat6	3	1	1	0
Pat7	1	0	0	0
Pat8	NA	0	NA	0
Pat9	0	0	0	0
Pat10	3	0	1	0
Pat11	5	1	1	0
Pat12	NA	NA	1	0
Pat13	2	0	0	0
Pat14	NA	NA	1	0
Pat15	NA	NA	1	0
Pat16	0	0	0	0
Pat17	NA	0	1	NA
Pat18	NA	NA	NA	NA
Pat19	NA	NA	NA	0
Pat20	3	0	1	0
Pat21	4	0	1	1
Pat22	2	0	1	0
Pat23	NA	0	NA	NA
Pat24	NA	0	0	0
Pat25	NA	NA	NA	0
Pat26	NA	NA	NA	NA
Pat27	NA	NA	0	NA
Pat28	NA	0	NA	0
Pat29	2	0	0	0
Pat30	NA	NA	NA	0

PatientNumber	AssociatedRiskFactorESBL	MultiDrugResistanceOrganismAntecedents	AntibioticTreatment6Months	NorfloxacineProphylaxis
Pat31	1	0	0	0
Pat32	1	0	0	0
Pat33	2	0	1	0
Pat34	NA	0	0	NA
Pat35	3	0	1	0
Pat36	NA	NA	NA	NA
Pat37	NA	NA	NA	0
Pat38	3	1	1	0
Pat39	0	0	0	0
Pat40	NA	0	NA	0
Pat41	2	0	1	0
Pat42	NA	NA	0	0
Pat43	NA	NA	NA	0
Pat44	0	0	0	0
Pat45	NA	NA	NA	0
Pat46	2	0	1	0
Pat47	NA	NA	NA	0
Pat48	NA	NA	NA	NA
Pat49	NA	NA	0	1

PatientNumber	RecentHospitalizationAntecedant	CountryBirthRisk	CountryBirth	BeforeHospitalizationOrigin	RecentTrip
Pat1	0	1	Morocco	Home	1
Pat2	0	0	France	Home	0
Pat3	1	0	France	Home	0
Pat4	0	0	France	Home	0
Pat5	0	0	France	Home	NA
Pat6	1	0	France	Home	0
Pat7	0	1	Conakry Guinea	Home	0
Pat8	1	1	Mauritius	Home	0
Pat9	0	0	France	Home	0
Pat10	0	1	Italy	Home	1
Pat11	1	1	Portugal	Home	1
Pat12	1	0	France	Home	0
Pat13	0	1	Mali	Hospitalization	1
Pat14	0	NA	NA	Home	0
Pat15	1	0	France	Hospitalization	0
Pat16	0	0	FRANCE	Home	0
Pat17	0	0	France	Home	0
Pat18	NA	1	India	Home	1
Pat19	NA	0	France	Hospitalization	0
Pat20	1	1	Senegal	Home	0
Pat21	1	1	SriLanka	Home	0
Pat22	0	1	Burkina Faso	Home	0
Pat23	0	1	Tunisia	Home	1
Pat24	NA	0	France	Home	0
Pat25	NA	0	France	Home	0
Pat26	NA		NA	Home	NA
Pat27	NA	0	France	Home	O
Pat28	0	0	France	Home	0
Pat29	1	1	Sri Lanka	Home	0
Pat30	1	1	Niger	Home	0

PatientNumber	RecentHospitalizationAntecedant	CountryBirthRisk	CountryBirth	BeforeHospitalizationOrigin	RecentTrip
Pat31	0	1	Morocco	Home	0
Pat32	1	0	France	Home	0
Pat33	1	0	France	Home	0
Pat34	0	0	France	Home	0
Pat35	1	1	Pakistan	Home	0
Pat36	NA	NA	NA	NA	NA
Pat37	1	NA	NA	Home	0
Pat38	1	0	France	Home	0
Pat39	0	0	France	Home	0
Pat40	0	1	Algeia	Home	0
Pat41	1	0	France	Hospitalization	0
Pat42	0	0	France	Home	0
Pat43	0	0	France	Hospitalization	0
Pat44	0	0	France	Home	0
Pat45	NA	0	France	Home	0
Pat46	1	0	France	Home	0
Pat47	NA	NA	NA	Home	0
Pat48	NA	NA	NA	NA	NA
Pat49	1	Serbia	Home	NA	0

PatientNumber	GNSepsisDuringHospitalization	ATBTeatmentHospitaliztion	Abundance	Observed	Shannon	ESBLPresence
Pat1	0	0	103	NA	NA	0
Pat2	0	0	48983	87	3.34	0
Pat3	0	1	155079	20	0.79	0
Pat4	0	0	NA	NA	NA	0
Pat5	0	0	71522	73	2.99	0
Pat6	0	1	11173	43	0.29	1
Pat7	0	0	46742	81	1.99	0
Pat8	0	0	NA	NA	NA	0
Pat9	0	0	38471	42	0.83	0
Pat10	0	0	26989	68	2.66	0
Pat11	0	1	3061	NA	NA	1
Pat12	1	1	122513	25	0.10	0
Pat13	0	0	21142	78	2.66	0
Pat14	0	0	11967	73	3.04	1
Pat15	0	1	15255	67	1.59	NA
Pat16	0	0	93393	66	2.64	0
Pat17	0	0	80166	80	3.10	0
Pat18	0	0	NA	NA	NA	1
Pat19	0	1	118481	74	2.63	0
Pat20	0	1	NA	NA	NA	0
Pat21	1	1	NA	NA	NA	1
Pat22	0	0	61498	74	3.19	0
Pat23	0	0	NA	NA	NA	NA
Pat24	0	0	41688	79	2.70	0
Pat25	0	1	NA	NA	NA	NA
Pat26	0	0	6524	NA	NA	0
Pat27	0	0	115355	69	2.81	1
Pat28	0	1	NA	NA	NA	0
Pat29	0	1	141262	20	0.55	0
Pat30	0	0	NA	NA	NA	NA

PatientNumber	GNSepsisDuringHospitalization	ATBTeatmentHospitaliztion	Abundance	Observed	Shannon	ESBLPresence
Pat31	0	0	52613	69	3.30	0
Pat32	0	1	NA	NA	NA	NA
Pat33	0	1	NA	NA	NA	0
Pat34	0	1	NA	NA	NA	0
Pat35	0	1	73593	57	2.46	0
Pat36	NA	NA	31360	76	2.44	0
Pat37	0	1	67222	71	2.37	1
Pat38	0	1	226	NA	NA	NA
Pat39	0	0	24478	69	2.02	0
Pat40	0	1	9	NA	NA	1
Pat41	0	1	2090	NA	NA	0
Pat42	0	0	30204	78	3.08	0
Pat43	0	1	153895	22	0.11	0
Pat44	0	0	121324	38	1.58	1
Pat45	0	1	NA	NA	NA	0
Pat46	0	1	71463	60	2.28	0
Pat47	0	0	40998	83	2.78	0
Pat48	NA	NA	120297	76	2.38	0
Pat49	1	18069	70	2.66	1.01	

Table S3. Nutrition score and BMI of 10 healthy volunteers.

Patient number	Category	Nutrtition score	Body mass index (BMI)	Age	Sex	Alcohol intake score
Pat50	Healthy	8.33	23.83	59	F	0
Pat51	Healthy	5.17	25.93	40	M	0
Pat52	Healthy	10.08	28.71	51	M	0
Pat53	Healthy	5.75	24.22	56	M	0
Pat54	Healthy	1.75	20.99	57	M	0
Pat55	Healthy	4.67	25.96	57	M	0
Pat56	Healthy	5.58	23.94	59	M	0
Pat57	Healthy	5.5	33.45	61	M	0
Pat58	Healthy	9	25.01	62	M	0
Pat59	Healthy	2.67	25.21	72	M	0
Pat60	Healthy	0.75	38.06	73	M	0

Table S4. Eigenvalues and amount of the variation explained by each principal component (PC) of the MCA analysis.

Dataset	Dimension	Eigenvalue	Variance (%)	Cumulative variance (%)
Clinical data	Dim1	0.657	43.780	43.780
	Dim2	0.369	24.583	68.362
	Dim3	0.254	16.931	85.294
	Dim4	0.093	6.222	91.515
	Dim5	0.077	5.114	96.629
	Dim6	0.051	3.371	100.000
Lifestyle data	Dim1	0.445	17.130	17.130
	Dim2	0.376	14.477	31.607
	Dim3	0.341	13.113	44.720
	Dim4	0.286	11.001	55.721
	Dim5	0.257	9.898	65.619
	Dim6	0.217	8.339	73.958
	Dim7	0.171	6.568	80.526
	Dim8	0.156	5.999	86.525
	Dim9	0.114	4.366	90.891
	Dim10	0.084	3.239	94.130
	Dim11	0.071	2.744	96.874
	Dim12	0.058	2.228	99.101
	Dim13	0.023	0.899	100.000

Table S5.Correlation ratios (eta-squared) of the main three dimensions of the MCA analysis.

		Dim1	Dim2	Dim3
Clinical data	CasControl	0.715	0.044	0.013
	ESBLRisk2	0.810	0.857	0.994
	AntibioticTreatment6Month	0.756	0.086	0.009
	ESBLPresence	0.346	0.488	0.000
Lifestyledata	CasControl	0.687	0.027	0.016
	SportScore2	0.516	0.535	0.264
	Shannon2	0.518	0.407	0.372
	NutScore2	0.273	0.417	0.454
	PrecariousnessScore2	0.233	0.496	0.599

Table S6. Most characteristic categories according to dimensions 1 and 2.

		Category	Estimate	p.value
**Clinical data**	**Dim1**	**Antibiotic intake**	0.706	3.061e-07
		**Decompensated**	0.692	1.402e-06
		**ESBLRisk(q4)**	1.203	2.812e-03
		**ESBL presence**	0.607	5.067e-03
		**ESBLRisk(q3)**	0.431	3.487e-02
		**ESBL absence**	-0.607	5.067e-03
		**ESBLRisk(q1)**	-1.137	2.668e-03
		**Compensated**	-0.692	1.402e-06
		**No antibiotic intake**	-0.706	3.061e-07
	**Dim2**	**ESBLRisk(q4)**	1.120	9.285e-04
		**ESBL prescence**	0.473	4.266e-03
		**ESBL absence**	-0.473	4.266e-03
		**ESBLRisk(q3)**	-0.947	3.240e-03
**Lifestyle data**	**Dim1**	**Decompensated**	0.608	5.636e-07
		**AlphaDiversity(q1)**	0.677	2.617e-03
		**SportScore(q1)**	0.217	1.098e-02
		**AlphaDiversity(q4)**	-0.475	4.602e-02
		**SportScore(q4)**	-0.780	4.107e-02
		**Compensated**	-0.608	5.636e-07
	**Dim2**	**PrecariousnessScore(q**	0.699	5.719e-04
		**NutScore(q3)**	0.542	8.469e-03
		**SportScore(q2)**	1.240	1.114e-02
		**NutScore(q4)**	-0.510	2.204e-02
		**SportScore(q4)**	-0.911	1.843e-02
		**AlphaDiversity(q1)**	-0.631	2.207e-03

Table S7: Peccentage of missing data

Category	Nutrition Score	Sport Score	Precariousness Score	Body Mass Index	Reason Admssion	Cirrhosis Type	Ascite Presence	Hepatic Encephalpathy	Gastrointestinal Haemorrhage
Decompensated	0.192	0.462	0.385	0.192	0.077	0.077	0.077	0.077	0.077
Compensated	0	0	0	0.087	0	0	0	0	0

Category	Low Prothrombin Rate	Diabete	Associated Risk Factor ESBL	MultiDrug Resistance Organism Antecedents	Antibiotic Treatment 6 Months	Norfloxacine Prophylaxis	Recent Hospitalization Antecedant	Country Birth Risk	Country Birth
Decompensated	0.154	0.077	0.577	0.462	0.423	0.115	0.192	0.115	0.115
Compensated	0.957	0.174	0.478	0.304	0.217	0.261	0.217	0.087	0.130

Category	Before Hospitalization Origin	Recent Trip	GN Sepsis During Hopitalization	ATB Teatment Hospitaliztion	Abundance	Observed	Shannon	Ratio GNB_GPB_DO	ESBL Presence
Decompensated	0.077	0.115	0.077	0.077	0.346	0.500	0.500	0.231	0.192
Compensated	0	0.0874	0	0	0.174	0.261	0.261	0.043	0.043

Table S8. Results of the regression analyses for the association between patient status and diet score

Decompensated patients *versus* healthy individuals

formula1: glm(status ~ DietScore, family = binomial(link = logit))

Coefficients:

	Estimate [95%CI]	Standard error	z value	pvalue
(Intercept)	-2.157 [-4.073; -0.825]	0.807	-2.672	0.0075
DietScore	0.381 [0.124; 0.748]	0.154	2.473	0.0134

formula2: glm(status ~ DietScore + DietScore:sex + DietScore:age, family = binomial(link = logit))

Coefficients:

	Estimate [95%CI]	Standard error	z value	pvalue
(Intercept)	-2.107 [-4.146; -0.762]	0.830	-2.540	0.0111
DietScore	3.274 [-20.347; NA]	287.947	0.011	0.9909
DietScore:age	-0.017 [-0.051; 0.007]	0.014	-1.282	0.1999
DietScore:sexM	-1.878 [NA; 56.602]	287.945	-0.007	0.9948

Compensated patients versus healthy individuals

formula1: glm(status ~ DietScore, family = binomial(link = logit))

Coefficients:

	Estimate [95%CI]	Standard error	z value	pvalue
(Intercept)	-1.390 [-2.922; -1.94]	0.676	-2.057	0.0397
DietScore	0.110 [-0.082; 0.331]	0.103	1.072	0.2237

formula2: glm(status ~ DietScore + DietScore:sex + DietScore:age, family = binomial(link = logit))

Coefficients:

	Estimate [95%CI]	Standard error	z value	pvalue
(Intercept)	-1.419 [- 3.130; -0.196]	0.696	-2.039	0.0415
DietScore	0.801 [-0.280; 2.043]	0.573	1.398	0.1621
DietScore:age	-0.013 [-0.034; 0.005]	0.010	-1.340	0.1802
DietScore:sexM	0.097 [-0.214; 0.488]	0.166	0.585	0.5588

Decompensated patients *versus* compensated patients

formula1: glm(status ~ DietScore, family = binomial(link = logit))

Coefficients:

	Estimate [95%CI]	Standard error	z value	pvalue
(Intercept)	-0.353 [-1.138; 0.372]	0.380	-0.928	0.3533
DietScore	0.162 [0.019; 0.328]	0.078	2.090	0.0366

formula2: glm(status ~ DietScore + DietScore:sex + DietScore:age, family = binomial(link = logit))

Coefficients:

	Estimate [95%CI]	Standard error	z value	pvalue
(Intercept)	-0.321 [- 1.112; 0.413]	0.383	-0.838	0.402
DietScore	0.880 [-0.660; 5.746]	1.150	0.766	0.444
DietScore:age	-0.001 [- 0.021; 0.019]	0.010	-0.128	0.898
DietScore:sexM	-0.668 [NA; 0.145]	0.928	-0.719	0.472

Table S9. Results of the regression analyses for the association between patient status microbiota diversity (OTU richness and Shannon index).

OTU richness

formula1: glm(status ~ richness, family = binomial(link = logit))

Coefficients:

	Estimate [95%CI]	Standard error	z value	pvalue
(Intercept)	3.388 [0.482; 7.714]	1.746	1.940	0.052
Richness	-0.058 [-0.120; -0.014]	0.026	-2.226	0.0260

formula2: glm(status ~ richness + richness:sex + richness:age, family = binomial(link = logit))

Coefficients:

	Estimate [95%CI]	Standard error	z value	pvalue
(Intercept)	3.611 [0.600; 8.174]	1.827	1.976	0.0481
Richness	-0.085 [-0.234; 0.034]	0.066	-1.302	0.1929
Richness:sexM	0.009 [-0.022; 0.054]	0.018	0.532	0.5947
Richness:age	0.0002 [-0.001; 0.002]	0.001	0.281	0.7789

Shannon index

formula1: glm(status ~ shannon, family = binomial(link = logit))

Coefficients:

	Estimate [95%CI]	Standard error	z value	pvalue
(Intercept)	2.850 [0.555; 6.369]	1.412	2.019	0.0435
Shannon	-1.410 [-2.823; -0.448]	0.579	-2.436	0.0148

formula2: glm(status ~ shannon + shannon:sex + shannon:age, family = binomial(link = logit))

Coefficients:

	Estimate [95%CI]	Standard error	z value	pvalue
(Intercept)	2.759 [0.485; 6.229]	1.393	1.981	0.0476
Shannon	-2.162 [-6.649; 1.030]	1.833	-1.180	0.2381
Shannon:sexM	0.206 [-0.675: 1.495]	0.503	0.410	0.6822
Shannon:age	0.009 [-0.039; 0.070]	0.026	0.360	0.7190

Table S10. Results of the regression analyses for the association between patient status and significant clinical and lifestyle variables.

Association between patient status and antibiotic intake
formula: glm(status ~ AntibioticTreatment, family = binomial(link = logit))

Coefficients:

	Estimate [95%CI]	Standard error	z value	pvalue
(Intercept)	1.386 [0.002; 3.277]	0.791	1.754	0.0795
AntibioticTreatment	-3.689 [-6.934; -1.451]	1.313	-2.809	0.0050

Association between patient status and associated risk factor ESBL
formula: glm(status ~ AssociatedRiskFactorESBL, family = binomial(link = logit))

Coefficients:

	Estimate [95%CI]	Standard error	z value	pvalue
(Intercept)	-4.116 [-10.010; -1.297]	2.026	-2.032	0.0422
AssociatedRiskFactorESBL	1.989 [0.684; 4.803]	0.937	2.123	0.0337

Association between patient status and microbiota diversity
formula: glm(status ~ Shannon, family = binomial(link = logit))

	Estimate [95%CI]	Standard error	z value	pvalue
(Intercept)	2.274 [-0.047; 5.629]	1.360	1.672	0.0945
Shannon	-1.531 [-3.013 -0.490]	0.612	-2.501	0.0124
